# Evaluating the Feasibility and Acceptability of a Mobile Health–Based Female Community Health Volunteer Program for Hypertension Control in Rural Nepal: Cross-Sectional Study

**DOI:** 10.2196/15419

**Published:** 2020-03-09

**Authors:** Zhao Ni, Namratha Atluri, Ryan J Shaw, Jingru Tan, Kinza Khan, Helena Merk, Yunfan Ge, Shrinkhala Shrestha, Abha Shrestha, Lavanya Vasudevan, Biraj Karmacharya, Lijing L Yan

**Affiliations:** 1 Duke University Durham, NC United States; 2 Duke Kunshan University Kunshan China; 3 Kathmandu University Kathmandu Nepal

**Keywords:** hypertension, female community health volunteers, mHealth

## Abstract

**Background:**

Hypertension is a major modifiable risk factor for cardiovascular disease, the world’s leading cause of death. The prevalence of hypertension is disproportionately higher in South Asian countries than in other regions of the world. Screening for hypertension in primary care settings remains a challenge in many South Asian countries, including Nepal. Nepal is located in the Himalayan Mountains region, posing significant geographical challenges for its rural citizens to access primary health care and service delivery. This barrier increases the costs and inconvenience for rural Nepalis to access hypertension screening and treatment. As a result, the prevalence of hypertension in Nepal tripled in the last 25 years to 22.4%-38.6%. Nepal’s Ministry of Health and Population relies on female community health volunteers to link health centers and communities to provide basic health services. Over 50,000 of these volunteers in Nepal have received basic health care training and are assigned to take care of maternal and child health. Due to limited health care resources, adopting new methods to control hypertension is an urgent need in Nepal. Several recent studies in Nepal have recommended extending the role of female community health volunteers to include hypertension management through blood pressure monitoring and home-based education.

**Objective:**

The goal of this study was to assess if a mobile health–based female community health volunteer approach of combining the traditional community health volunteer program with digital technologies would be feasible and acceptable in rural Nepal.

**Methods:**

In this study, we recruited 17 female community health volunteers and extended their role from maternal and child health to hypertension management through screening blood pressures.

**Results:**

All 17 female community health volunteers successfully measured 1113 rural Nepalis’ blood pressures, identified 169 hypertensive patients, and collected health behaviors data of the 169 hypertensive patients. Among the 169 patients, 70% of them had a mobile phone, and 92% were interested in receiving health-related information via a mobile phone. Among those who were interested in receiving information via a mobile phone, 84% preferred voice calls, and 7% and 1% preferred texting and apps, respectively.

**Conclusions:**

Results from this study indicate that a digital health intervention that leverages feature-phones combined with female community health volunteers may be an acceptable and pragmatic way to implement an evidence-based program to reduce hypertension in rural Nepal.

## Introduction

Hypertension is a major modifiable risk factor for cardiovascular disease [1]. According to the Global Status Report on noncommunicable diseases, in the year 2010 alone, 9.4 million people died due to hypertension-related complications [2]. Compared to other regions of the world, the prevalence of hypertension is disproportionately higher in South Asian countries, such as Nepal. In Nepal in particular, the prevalence of hypertension tripled in the last 25 years to 22.4%-38.6% [3]. The mortality rate of hypertension has been steadily increasing in parallel, from 135.6 to 145.2 per 100,000 people from 1995 to 2015 [4]. Hypertension and related complications are considered major contributors to death and disability in Nepal [5]. Although hypertension can be effectively lowered using medication, there are several challenges to solving this issue in Nepal. First, there is inadequate screening for hypertension in primary care settings. Second, Nepal is mainly located in the Himalayan Mountains region, posing significant geographical challenges in access to primary health care and service delivery, which contributes to a high financial and human resource burden. Finally, among individuals identified as hypertensive, adherence to antihypertensive medication is consistently reported to be low, thereby increasing the risk of uncontrolled blood pressure and its complications [6].

Due to limited health care resources, adopting new methods to control hypertension is an urgent need in Nepal [7,8]. Nepal’s Ministry of Health and Population relies on female community health volunteers to link health centers and communities to provide basic health services [9]. Over 50,000 female community health volunteers in Nepal have received basic health care training and are assigned to take care of maternal and child health [10]. Several studies in Nepal have recommended extending the role of these volunteers to include hypertension management through blood pressure monitoring and home-based education [9-11]. This recommendation is based on the fact that, in Nepal, there is a lack of linkage to primary care among patients with hypertension. Due to the high geographic barriers in the Himalayas, an affordable means to communicate and provide ready access to hypertension care delivery is greatly needed. Because mobile phones are widely used in Nepal, with a household subscription level of over 75% and steadily increasing [12], they may provide the needed communication platform. It is highly possible that female community health volunteers could use mobile phones to collect hypertension data and link patients to primary care. This suggests combining the traditional community health volunteer program with an evidence-based hypertension reduction program and digital technologies could be a pragmatic way to reduce hypertension. The goal of this study was to assess if a mobile health (mHealth)–based female community health volunteer approach of combining the traditional volunteer program with digital technologies would be feasible and acceptable in rural Nepal.

## Methods

### Study Design and Participants

**Figure 1:**
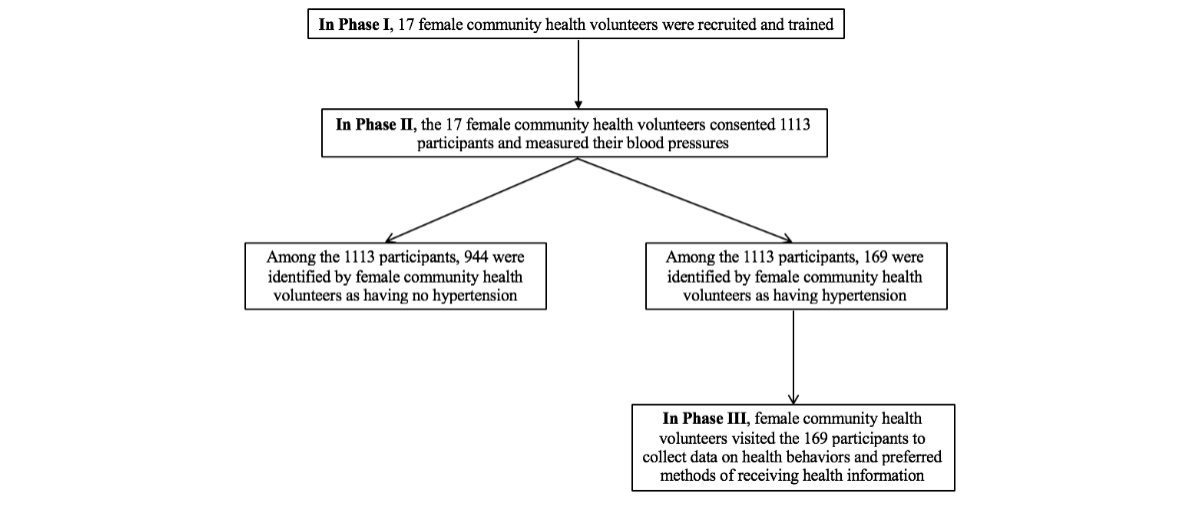
Study flow chart.

This study was approved by the Duke University Health System Institutional Review Board (Pro00092469), and the ethics review committee in Nepal (Reg. no. 83/2018). This entire study was conducted in three phases (see Figure 1). In Phase I, we recruited 17 female community health volunteers from two rural communities, nine in Dhunkharka and eight in Panchkhal, Nepal, to learn how to accurately measure blood pressure. The government of Nepal granted permission to approach the 17 female community health volunteers for this study. All 17 female community health volunteers provided informed consent for the study. Each volunteer was responsible for the wards assigned by the Nepal Ministry of Health and Population. The two rural communities were located about 30 miles away from the capital city, Kathmandu. Two local Nepali graduate students who were fluent in English and Nepali served as translators for this study. Our team organized two equivalent training sessions taught by two licensed physicians.

Each training session included three subsessions: a 20-minute hypertension education session, a 10-minute question and answer session, and a 30-minute data-collection training session. Training content was developed specifically for this study by our study coordinator Jingru Tan and the two physicians by adapting content from the World Health Organization’s Protocol titled “Prevention of Heart Attacks, Strokes, and Kidney Disease through Integrated Management of Diabetes and Hypertension” [13]. This protocol was part of the World Health Organization’s package of essential noncommunicable disease interventions for primary health care in low-resource settings. In the training sessions, the female community health volunteers learned and practiced measuring, reading, and documenting blood pressure through an electronic blood pressure cuff. The volunteers also practiced measuring blood pressure on each other and received feedback from physicians. After the training sessions, our research team evaluated each community health volunteer’s knowledge and practical skills on blood pressure assessment by assigning two team members to accompany each volunteer for half a day to observe their performance at measuring blood pressures. The two research team members evaluated the female community health volunteer’s performance using the blood pressure–measuring guideline retrieved from the “Seventh Report of the Joint National Committee on Prevention, Detection, Evaluation, and Treatment of High Blood Pressure” [14], which listed detailed information on how to measure blood pressure. After the observation, the two members provided feedback to each volunteer based on the blood pressure–measuring guideline and ensured that their skills for measuring blood pressure satisfied the guideline.

In Phase II, each female community health volunteer visited the rural residents in their respective wards and introduced this study. The inclusion criteria of participants were: (1) living in either of the two rural communities located in Dhunkharka and Panchkhal, Nepal for more than five years; (2) being able to speak Nepali or English; and (3) being 40 years old or older [13]. For those residents who provided written consent, the female community health volunteers measured their blood pressure twice on the right upper arm after 10 minutes of rest in a seated position at their homes, with feet on the floor and arm supported at heart level [14]. In total, 1159 residents satisfied the inclusion criteria, and 1113 of them were screened for hypertension, resulting in a recruitment rate of 96%. Of the 46 residents who refused to participate, 45 refused due to their busy schedules with farm work, and one refused without providing a specific reason. Participants with hypertension were selected for a survey in Phase III. In Phase III, female community health volunteers revisited the selected hypertensive participants, measured their blood pressure, and asked them to complete a 15-item survey. Each survey took about 15 minutes to complete.

### Data Collection and Variables

In Phase II, volunteers collected participants’ demographic variables, including gender, age, educational level, and health outcomes, including systolic blood pressure, diastolic blood pressure, and heart rate. The female community health volunteers visited each participant and measured blood pressure twice on the right upper arm after 10 minutes of rest in a seated position [15] by using an electronic blood pressure cuff (Omron HEM-7124; Omron Dalian Co. Ltd, China). The second measure was taken 5 minutes after the first measure. If the difference between the two measures was greater than 10 mmHg for systolic or diastolic blood pressure, a third measure was taken. The Omron blood pressure cuff has been widely used in Nepali clinical settings and is suitable for Nepali adults aged 18 years old or older. To ensure accuracy of measuring blood pressures, we used Omron blood pressure cuffs that have a bladder encircling at least 80 percent of the upper arm of an adult [14]. This Omron blood pressure cuff was a single size, but the selection of this cuff was based on its wide use in Nepali clinical settings and suitability for Nepali adults over 18 years of age [16,17]. The female community health volunteers can wrap the blood pressure cuff based on the upper arm circumference of a participant. In this study, we used the US Joint National Committee [14] definition of hypertension, in which hypertension is defined as average systolic blood pressure (SBP) equal to or greater than 140 mmHg, or an average diastolic blood pressure (DBP) equal to or greater than 90 mmHg [15,18]. The second visit in Phase III was to collect data on self-reported health behaviors, including smoking, alcohol consumption, medication adherence, hypertension awareness, phone use, and a preferred method of receiving health information (see Figure 1). The 15-item survey was adapted from the Morisky Green Levine Scale [19,20] and a 21-item survey querying from Johns Hopkins University School of Medicine [21].

### Outcomes and Metrics

The primary outcome of this study was an assessment of the feasibility of extending the role of female community health volunteers to blood pressure monitoring. Feasibility was documented by the assignment completion rate, defined as the number of community health volunteers who completed all three phases. Secondary outcomes included systolic blood pressure, diastolic blood pressure, and preference of mHealth intervention. We also assessed the acceptability of this mHealth program, defined as the number of participants who consented to receive health-related information via cell phone, compared to the total number of participants approached in Phase III.

### Statistical Analysis

All statistical analyses were performed using SAS 9.4 (SAS Institute, Cary, North Carolina, United States). Categorical variables included gender, educational level, marital status, smoking, drinking consumption, and use of antihypertensive medications. Numerical variables included age, height, weight, systolic blood pressure, diastolic blood pressure, and heart rate. Descriptive statistics, frequency (n), and percentage (%) of female community health volunteers who completed all three phases, and participants who consented to receive health-related information via cell phone, were reported to detail the feasibility of extending the role of female community health volunteers to blood pressure monitoring, and the acceptability of this mHealth program among participants. The prevalence of hypertension, rate of medication adherence, percentage of mobile phone users, awareness of hypertension, and interest in receiving health-related information via mobile phones were also studied.

## Results

The study sample included 1113 rural Nepalis with a mean age of 56.3 years old (SD 13.3), with a roughly equal distribution of male and female participants. Their ages ranged from 40-104 years old. Most participants (62%) had not received any formal education, and less than 6% had received higher education. Nearly one out of four participants (24%) had been diagnosed with hypertension by a doctor before the female community health volunteers measured their blood pressures; however, only 17% were taking medication to treat hypertension (Table 1). Therefore, among the participants who were diagnosed with hypertension by doctors, the percentage of them that were taking medications to treat hypertension was 69%.

Among the 1113 participants, 169 were identified by female community health volunteers to have hypertension in Phase II. The 169 participants included new hypertensive patients and hypertensive patients diagnosed by doctors who did not control their blood pressures within a normal range. The number of participants who were diagnosed and optimally treated was 189. Adding this number to the 169 hypertensive participants identified by the female community health volunteers, the total number of hypertensive participants was 358 (32%). The age range of the 169 participants was 40-104 years old. Their average systolic and diastolic blood pressures were 147 mmHg and 96 mmHg, respectively. Based on international hypertension guidelines [14,22], we categorized the 169 hypertensive participants into grade 1 (140≤SBP<160 mmHg and 90≤DBP<100 mmHg), and grade 2 (SBP≥160 mmHg and DBP≥100 mmHg). We found that 96 (57%) participants were in grade 1 and 73 (43%) were in grade 2. Among those 169 hypertensive rural Nepalis, 52% did not know that they had hypertension and 71% did not receive any treatment, such as medications to control their high blood pressure (Table 2). In terms of mobile phone usage, 70% of the hypertensive participants had a mobile phone, and 92% were interested in receiving health-related information via a mobile phone. Among those who were interested in receiving information via a mobile phone, 84% preferred voice calls, and 7% and 1% preferred texting and apps, respectively. All 17 female community health volunteers completed the three phases and returned their documented participants’ blood pressures to us, suggesting the high feasibility of this approach. The acceptability was high, with 92% of the participants willing to receive health-related information via cell phone.

**Table 1:** Baseline characteristics of the sample in Phase II at enrollment.

Variable		All participants	Dhunkharka	Panchkhal	*P* value
Number of participants, n (%)		1113 (100)	594 (53.4)	519 (46.6)	
**Gender^a^, n (%)**					**.49**
	Male	520 (46.7)	283 (47.6)	237 (45.7)	
	Female	592 (53.2)	310 (52.2)	282 (54.3)	
**Education^b^, n (%)**					**.08**
	No education	685 (61.5)	355 (59.8)	330 (63.6)	
	Primary education	267 (24.0)	155 (26.1)	112 (21.6)	
	Secondary education	92 (8.3)	41 (6.9)	51 (9.8)	
	Higher education	64 (5.8)	38 (6.4)	26 (5.0)	
**Had been diagnosed with hypertension by a doctor^c^, n (%)**					**.002**
	No	842 (75.7)	428 (72.1)	414 (79.8)	
	Yes	268 (24.1)	165 (27.8)	103 (19.8)	
**Had been taking antihypertensive^d^, n (%)**					**<.001**
	No	920 (82.7)	468 (78.8)	452 (87.1)	
	Yes	185 (16.6)	125 (21.0)	60 (11.6)	
Age (Year), mean (SD)		56.3 (13.3)	55.5 (12.4)	57.3 (14.2)	.03

^a^The Gender information of one participant from Dhunkharka was missing.

^b^The Education information of five participants from Dhunkharka was missing.

^c^In Dhunkharka and Panchkhal, one and two participants’ information on whether they had been diagnosed with hypertension by a doctor was missing, respectively.

^d^In Dhunkharka and Panchkhal, one and seven participants’ information on whether they had been taking antihypertensives was missing, respectively.

**Table 2:** Baseline characteristics of the sample selected into Phase III.

Variable		All participants	Dhunkharka	Panchkhal	*P* Value
Number of participants, n (%)		169 (100)	68 (40.2)	101 (59.8)	—^a^
**Gender, n (%)**					**.49**
	Male	99 (58.6)	42 (61.8)	57 (56.4)	
	Female	70 (41.4)	26 (38.2)	44 (43.6)	
**Education, n (%)**					**.13**
	No education	99 (58.6)	35 (51.5)	64 (63.4)	
	Primary education	46 (27.2)	23 (33.8)	23 (22.8)	
	Secondary education	10 (5.9)	3 (4.4)	7 (6.9)	
	Higher education	11 (6.5)	7 (10.3)	4 (4.0)	
**Had been diagnosed with hypertension by a doctor (received prescription of antihypertensive medications), n (%)**					**.03**
	No	88 (52.1)	42 (61.8)	46 (45.5)	
	Yes	79 (46.7)	25 (36.8)	54 (53.5)	
**Had been taking antihypertensive, n (%)**					**.01**
	No	120 (71.0)	56 (82.4)	64 (63.4)	
	Yes	45 (26.6)	11 (16.2)	34 (33.7)	
**Stopped taking medication when felt worse^b^, n (%)**					**.01**
	No	41 (91.1)	8 (72.7)	33 (97.1)	
	Yes	3 (6.7)	3 (27.3)	0 (0)	
**Stopped taking medication when felt better^b^, n (%)**					**.05**
	No	39 (86.7)	8 (72.7)	31 (91.2)	
	Yes	5 (11.1)	3 (27.3)	2 (5.9)	
**Perspective on whether hypertensive patients need to take antihypertensive daily, n (%)**					**.01**
	No	37 (21.9)	22 (32.4)	15 (14.9)	
	Yes	114 (67.5)	39 (57.4)	75 (74.3)	
**Had smoked before, n (%)**					**.57**
	No	81 (47.9)	31 (45.6)	50 (49.5)	
	Yes	82 (48.5)	35 (51.5)	47 (46.5)	
**Current smoker^c^, n (%)**					**.80**
	No	30 (36.6)	14 (40.0)	16 (34.0)	
	Yes	39 (47.6)	17 (48.6)	22 (46.8)	
**Drinking alcohol, n (%)**					**.03**
	Never	105 (62.1)	38 (55.9)	67 (66.3)	
	Drink during events	31 (18.3)	12 (17.6)	19 (18.8)	
	1-2 times a month	6 (3.6)	3 (4.4)	3 (3.0)	
	1-2 times a week	4 (2.4)	2 (2.9)	2 (2.0)	
	Daily	15 (8.9)	12 (17.6)	3 (3.0)	
**Had a cell phone, n (%)**					**.64**
	No	44 (26.0)	16 (23.5)	28 (27.7)	
	Yes	119 (70.4)	48 (70.6)	71 (70.3)	
**The phone is a smartphone^d^, n (%)**					**.39**
	No	81 (68.1)	34 (70.8)	47 (66.2)	
	Yes	33 (27.7)	11 (22.9)	22 (31.0)	
**Interested in receiving health-related information, n (%)**					**.01**
	No	7 (4.1)	6 (8.8)	1 (1.0)	
	Yes	156 (92.3)	59 (86.8)	97 (96.0)	
**Preferred methods,^e^ n (%)**					**.13**
	Voice call	131 (84.0)	50 (84.7)	81 (83.5)	
	Text message	11 (7.1)	2 (3.4)	9 (9.3)	
	Apps	2 (1.3)	2 (3.4)	0 (0)	
	Don’t know	1 (0.6)	0 (0)	1 (1.0)	
Weight (kg), mean (SD)		60.9 (12.5)	58.4 (10.5)	62.8 (13.6)	.05
Height (cm), mean (SD)		159.7 (11.0)	157.6 (14.7)	160.7 (8.6)	.20
Age (years), mean (SD)		59.3 (13.3)	57.0 (10.7)	60.8 (14.7)	.07
BMI (kg/m^2^), mean (SD)		24.6 (5.7)	24.3 (5.4)	24.8 (5.9)	.71

^a^Not applicable.

^b^Answers were from participants who had been taking antihypertensive.

^c^Answers were from participants who had smoked before.

^d^Answers were from participants who had a cell phone.

^e^Answers were from participants who had interest in receiving health-related information.

## Discussion

### Primary Findings

This study demonstrates high feasibility and acceptability of using female community health volunteers, an integral component of the Nepali health care infrastructure, in collecting blood pressure data. Although female community health volunteers in Nepal have mainly been used for maternal and child health interventions, our study shows that these volunteers can be trained to assist with other important health initiatives. In addition to quickly learning how to use electronic blood pressure cuffs and record and interpret blood pressure readings, the female community health volunteers were also keen on imparting their newly gained knowledge about hypertension to their community members. Globally, the role of community health workers (CHWs) has shifted from serving a specific disease or population, such as maternal and child health, to help solve community health problems that are the most emergent or need the most resources [23]. Hypertension is a major risk factor for cardiovascular disease, the world’s leading cause of death. Bone et al [24] and Krieger et al [25] have extended the role of a CHW to include controlling high blood pressure and demonstrated that CHWs could successfully increase follow-up care for hypertensive patients. Our findings are aligned with the existing evidence. Shifting primary care duties of managing hypertension in low- and middle- income countries from physicians to nonphysician health care workers, such as CHWs, has been tested in studies and showed potential in reducing blood pressure [26]. For example, a high-quality randomized trial conducted in rural China and India [27] found that CHWs using an Android-powered app could reduce blood pressure and improve antihypertensive medication adherence among rural residents with cardiovascular disease. Studies conducted in Pakistan [28], Bangladesh, and Sri Lanka [29] also found the same result that CHW-led interventions can help reduce blood pressure.

The results from this study indicate that hypertension management remains grossly inadequate in rural Nepal. Among the 169 female community health volunteer–identified hypertensive participants, 52% were not aware of their hypertension and nearly 71% did not regularly take medications to control their hypertension. Overall, 22% of the participants were not aware that hypertensive patients need to take antihypertensive medications to manage high blood pressure. This data reveals how uninformed participants are about their blood pressure, how to manage it through medication adherence, and the risks involved with living with this chronic illness. Medication adherence can be jointly influenced by external factors such as health systems, health providers, and access to care, and intrinsic factors associated with patients. Intrinsic nonadherence is caused primarily by forgetfulness, misunderstanding of the medication regimens, and lack of communication with health workers [30,31]. While several medication adherence–increasing interventions exist [32], most are ill-suited for low-income countries lacking necessary health care infrastructure [6].

In this study’s participant population, in addition to the financial disadvantage indicated by their reported income levels, participants also faced geographic isolation. We found through our study that participants’ access to primary health care facilities is very limited, which can contribute to their lack of understanding of how to self-manage their health and the consequences of poor medication adherence. Due to this inconsistent relationship with the health care system, many rural Nepalis in our study also express distrust of medical professionals and are less inclined to seek out health services on their own. However, the female community health volunteers are highly trusted throughout their communities and have close relationships with residents. This study has shown that the female community health volunteers can implement hypertension-based health initiatives; thus, future interventions to reduce hypertension in Nepal could leverage female community health volunteers for community-based blood pressure monitoring. In this study, the electronic blood pressure cuffs that we used were powered by batteries, which were affordable and accessible in rural Nepal. For example, a set of four regular batteries was 80 Nepalese Rs (US $0.70). Today, there are many types of blood pressure monitors available in the market. For instance, those with a USB connection can be charged by portable batteries and can provide users the guidance on acceptable blood pressure ranges. They can be adapted in rural Nepal by female community health volunteers too.

The collected data also revealed that 70% of participants owned a feature-phone that had text messaging abilities. Overall, 84% of the participants also preferred receiving health information through voice calls or voice messages on their phones Considering the huge cost barrier to improving health outcomes in rural Nepal, both from an individual patient perspective and a governmental perspective, these data suggest a new avenue to deliver care by utilizing mobile health technologies [33,34]. Since lack of awareness about health information and insufficient medication adherence were shown to be problematic for hypertensive patients in rural Nepal, the high prevalence of feature-phone use and patients’ interest in getting health information via voice calls provide a strong rationale to support a feature phone-based software application. Integrating such an intervention with existing infrastructure, such as the female community health volunteer system, appears promising as the volunteers are already highly trusted and have a wide reach. For example, after visiting a hypertensive resident, the female community health volunteer may use phone calls to follow up on the resident’s access to antihypertensive medications. When refilling medications is necessary, the female community health volunteers can bring medications to that resident in their next visit. Also, the cell phones may be used to facilitate female community health volunteers to monitor rural residents’ blood pressures and thus increase early diagnosis of hypertension. For example, by following up on residents and asking about their previously tested blood pressures (tested in a clinic or hospital) via cell phones, the volunteers could find out if the residents’ blood pressures had increased. If a resident’s blood pressure is increasing, the female community health volunteers can visit that resident and measure his or her blood pressure to confirm whether the resident is hypertensive.

The technology considered in this feature phone–based intervention should refer to basic mobile phones, which will enable broader coverage in low-income countries such as Nepal. These devices lack smartphone capabilities and tend to have custom-designed software and user interfaces. Many studies have confirmed the effectiveness and affordability of text message reminders to improve adherence to medication intake and to encourage regular visit attendance to manage chronic diseases [35]. Also, Nepal’s ministry of health has prioritized mHealth-led interventions by releasing “National e-health strategies,” aiming to strengthen the application of information and communications technologies in support of health and health-related fields [36]. When assessed together, the aforementioned factors demonstrate an intervention to reduce hypertension that leverages feature-phones as the communication platform could possibly be a well-timed program for Nepal.

While this study contributes important knowledge to the use of mHealth in rural Nepal, it has several limitations. First, the female community health volunteers identified hypertensive participants by only a single visit. Given the high variability in blood pressure, ideally, the volunteers should visit participants twice to measure their blood pressure. Second, in this study, variables such as weight and drinking alcohol were not measured by female community health volunteers but were self-reported by participants. Also, a challenge in the management of chronic conditions, such as hypertension and diabetes, in Nepal is access to medications and a patient’s ability to pay for them. In this study, we did not address this issue. Finally, our analyses were based on the 169 hypertensive patients identified by the female community health volunteers but did not include those diagnosed by doctors and optimally controlled their blood pressures within the normal range.

### Conclusion

Given the large burden of hypertension in Nepal, adopting new methods to control hypertension has become an emergent need for rural Nepalese. Results from this study indicate that a mobile health intervention that leverages feature-phones combined with female community health volunteers is a new way to implement an evidence-based program to reduce hypertension in rural Nepal. More future research should be conducted to test the feasibility and acceptability of such programs.

## Abbreviations

CHW: community health worker
DBP: diastolic blood pressure
mHealth: mobile health
SBP: systolic blood pressure
