# Correction: Evaluating the Feasibility and Acceptability of a Mobile Health–Based Female Community Health Volunteer Program for Hypertension Control in Rural Nepal: Cross-Sectional Study

**DOI:** 10.2196/19048

**Published:** 2020-06-11

**Authors:** Zhao Ni, Namratha Atluri, Ryan J Shaw, Jingru Tan, Kinza Khan, Helena Merk, Yunfan Ge, Shrinkhala Shrestha, Abha Shrestha, Lavanya Vasudevan, Biraj Karmacharya, Lijing L Yan

**Affiliations:** 1 Duke University Durham, NC United States; 2 Duke Kunshan University Kunshan China; 3 Kathmandu University Kathmandu Nepal

The authors of “Evaluating the Feasibility and Acceptability of a Mobile Health–Based Female Community Health Volunteer Program for Hypertension Control in Rural Nepal: Cross-Sectional Study” (JMIR Mhealth Uhealth 2020;8(3):e15419) noticed a typo in the Abstract of their published manuscript.

The following sentence:

The goal of this study was to assess if a mobile health–based female community health volunteer approach of combining the traditional community health volunteer program with digital technologies would be feasible and acceptable in rural Nepa

Has been revised to:

The goal of this study was to assess if a mobile health–based female community health volunteer approach of combining the traditional community health volunteer program with digital technologies would be feasible and acceptable in rural Nepal.

The correction will appear in the online version of the paper on the JMIR website on June 11, 2020, together with the publication of this correction notice. Because this was made after submission to PubMed, PubMed Central, and other full-text repositories, the corrected article has also been resubmitted to those repositories.

